# Investigation by Digital Image Correlation of Mixed-Mode I and II Fracture Behavior of Polymeric IASCB Specimens with Additive Manufactured Crack-Like Notch

**DOI:** 10.3390/ma14051084

**Published:** 2021-02-26

**Authors:** Tommaso Maria Brugo, Ivo Campione, Giangiacomo Minak

**Affiliations:** 1 Department of Industrial Engineering (DIN), Alma Mater Studiorum—Università di Bologna, Viale Del Risorgimento 2, 40136 Bologna, Italy; tommasomaria.brugo@unibo.it; 2 Department of Industrial Engineering (DIN), Alma Mater Studiorum—Università di Bologna, Via Fontanelle 40, 47121 Forlì, Italy; ivo.campione@unibo.it

**Keywords:** additive manufacturing, selective laser sintering, polyamide, nylon, fracture toughness, J-integral, mixed-mode I-II, IASCB specimens, digital image correlation

## Abstract

In this work, the fracture mechanics properties of polyamide (PA) specimens manufactured by the selective laser sintering (SLS) technology are investigated, in which an embedded crack-like notch was inserted in the design and produced during the additive manufacturing (AM) phase. To cover a wide variety of mode I/II mixity levels, the inclined asymmetrical semicircular specimen subjected to three points loading (IASCB) was employed. The investigation was carried out by analyzing the full displacement field in the proximity of the crack tip by means of the digital image correlation (DIC) technique. To characterize the material, which exhibits a marked elastic-plastic behavior, the quantity J-integral was evaluated by two different methods: the first one exploits the full fields measured by the DIC, whereas the second one exploits the experimental load–displacement curves along with FEM analysis. The DIC methodology was experimentally validated and proposed as an alternative method to evaluate the J-integral. It is especially suited for conditions in which it is not possible to use the conventional LDC method due to complex and possibly unknown loading conditions. Furthermore, results showed that the AM technique could be used effectively to induce cracks in this type of material. These two aspects together can lead to both a simplification of the fracture characterization process and to the possibility of dealing with a wider number of practical, real-world scenarios. Indeed, because of the nature of the additive manufacturing process, AM crack-like notches can be sintered even having complex geometry, being three-dimensional and/or inside the tested structure.

## 1. Introduction

Selective laser sintering (SLS) is a very popular and widespread technology among additive manufacturing, which is nowadays exploited in many different fields, from industrial design to architecture or medical devices. It enables the manufacture of complex geometries, which would be hard or even impossible to obtain through traditional subtractive techniques. The working principle of the SLS is based on the use of a laser to sinter powder layer-by-layer from the bottom-up, in this way, building the object section-by-section. One of its advantages is the capability of processing a large variety of materials [1], such as metals, polymers, ceramics or wax. One drawback, however, is that the mechanical properties of the sintered product are anisotropic, depending on the direction in which the geometry is built up. As a consequence, the buildup direction is to be carefully chosen and becomes an important process parameter, in particular when dealing with the design of components characterized by their structural properties [2]. In this work, the fracture mechanics properties of polyamide (PA), sintered via the selective laser sintering (SLS) technology, are investigated. The specimens have the peculiarity of being sintered with embedded crack-like notches (sharp notches). The advantage of directly sintering artificial crack is that this way, there is very little limitation in the position and orientation of the crack-like notch to manufacture, differently from the case in which traditional manufacturing methods are used. This allows one to be able to tackle even complex three-dimensional fracture mechanical problems. The possibility of sintering artificial crack-like notches during the SLS process was investigated by T. Brugo et al. [3], showing the feasibility of the approach. The same method was then successfully applied in [4] to study the fracture behavior of high-strength metallic material. A similar approach was also successfully applied to predict fracture loads of 3D-Printed ABS specimens, in which a pre-crack is embedded in the additive manufacturing process, this time using fused-deposition modeling (FDM) technology [5].

In the literature, two main types of experimental setups can be found to investigate the fracture behavior of mode I/II mixity: the first one is based on asymmetrical rectangular specimens, such as [6,7], the modified compact tension (CT) specimen [8] or the mixed-mode bending (MMB) test [9]. This latter method allows one to cover a spectrum of different mode ratios by varying the length of the loading lever. The second one exploits disc-like specimens, such as the Brazilian disc (BD) [10], the semicircular bend (SCB) [11] specimen, the asymmetric semicircular bend (ASCB) [12,13] specimen and the Inclined edge-cracked semicircular bend (IASCB) [14,15] specimen. In this study, the IASCB specimen subjected to three-point bend loading (the test configuration is described in detail in Section 2.2) was used. This setup permits to study of a large range of mode I/II mixity levels without requiring an elaborate loading fixture. The IASCB specimens were sintered with different crack-like notch angles and tested on different support spans. In three of them (for pure mode I), the crack was manually induced, with the scope of carrying out a comparison with the ones with the sintered crack-like notch.

The digital image correlation technique (DIC) was exploited to determine the fracture behavior of the material. Together with the electronic speckle pattern interferometry (ESPI) [16], the DIC is one of the most popular optical techniques used to evaluate the full displacement and strain field [17]. The main advantage of the DIC is that it only requires cameras of adequately high resolution and an algorithm to perform the correlation between the images, which is carried out by exploiting a speckle pattern (usually spray-painted) on the surface of the sample. This pattern serves as an aid for the correlation algorithm, which is based on matching subsets of corresponding pixels in consecutive images. Consequently, the relative displacement may be measured and thus be used to quantify the deformation. In [18,19], the DIC technique was used to investigate and characterize the fracture and fatigue behavior of a brittle material, namely the asphalt concrete, by exploiting the SCB test configuration. The DIC system used in [19] has the peculiarity of being composed of two cameras, which makes it a stereo DIC system.

In the case study, since the specimens exhibit a strong elastic-plastic behavior, the J-integral was chosen as the fracture characterization parameter. In fact, the stress intensity factors (SIFs) are suitable parameters only in the case of material that can be considered linear elastic. The J-integral, conversely, can be successfully used to characterize the fracture mechanics behavior of elastic-plastic material, such as a wide range of tough polymers, as stated in the standard ASTM D6068-96 [20]. The J-integral was originally proposed by Rice [21] and represents the strain energy release rate or energy per unit fracture surface area of a material. It is a common parameter to characterize near-crack-tip deformation filed in elastic and elastic-plastic material. Usually, the J-integral is calculated from the integration of the experimental load–displacement curve (henceforth named LDC), knowing the geometry factors previously obtained by finite element analysis (FEM). This procedure is usually adopted in standards such as the ASTM D6068-96 for CT and SEB specimens [20]. However, this technique is suitable only for laboratory tests in which the geometry factors for the specific specimen geometry are previously known, and the loading-displacement curve can be measured. Conversely, on a complex structure under operating conditions, it is difficult or even impossible to compute the geometrical factors and the load–displacement boundary conditions for the specific spot in which the crack was nucleated. An alternative method consists of computing the J-integral from the displacement fields around the crack tip, measured by DIC. The main advantage of this technique is that it can be performed directly on the structure, without the need of previously knowing neither the geometrical factors nor the loading boundary conditions. The DIC-based methodology consists of numerically computing the J-integral along a rectangular contour of the DIC grid [22,23,24,25,26]. For the J-integral calculation, displacement, strain and stress maps are needed. The displacement field is obtained directly from the DIC; the strain field can be computed by numerical differentiation of the former, whereas the stress maps can be computed from the strain maps, given that the constitutive equations of the material are known. The material has thus to be previously characterized, for example, in terms of Ramberg–Osgood parameters, as done for instance in [23]. The J-integral values calculated by the DIC-based methodology were finally compared to the ones obtained employing the LDC method.

## 2. Materials and Methods

### 2.1. Specimen Fabrication

The specimens were produced by an EOS Formiga P100 selective laser sintering (SLS) machine (EOS GmbH, Krailling, Germany), using fine polyamide PA 2200 powder purchased from Prototal Plastic Design and Service (Götene, Sweden), the mechanical properties experimentally determined by three-point bending tests are shown in Table 1. The laser-sintered specimens were manufactured from mixed powder, recycled and new in equal proportions. During the fabrication via the SLS technology, the specimens were oriented with the thickness in the built direction.

### 2.2. IASB Specimens

**Figure 1:**
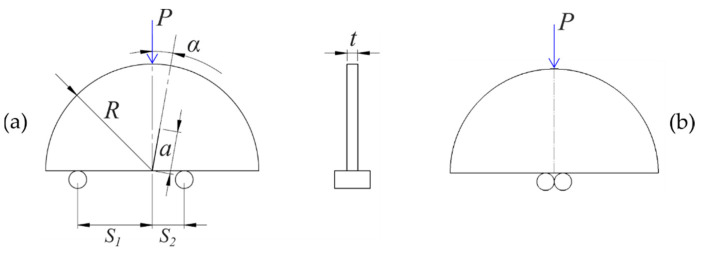
Geometrical features and loading conditions of the inclined asymmetrical semicircular specimen subjected to three points loading (IASCB) specimen; (**a**) fracture test; (**b**) indentation test.

The IASCB specimen is a semicircular disk of radius *R* and thickness *t* with an embedded radial edge-crack of length *a*, tilted with respect to the load direction of an angle *α*. The fracture test consists of the application of a vertical force *P*, with the specimen located on two bottom supports, as shown in Figure 1a. By varying some geometrical parameters, such as the support spans *S*_1_ and *S*_2_ and the crack tilt angle *α*, it is possible to obtain a large variety of mixed I/II modes. The values of *t*, *R,* and *a* were identical for all the specimens (*t =* 6 mm, *R* = 60 mm, *a* = 24 mm), and *S_1_* was kept fixed at 42 mm. The different mode mixity was obtained by choosing the suitable combination of *S_2_*/*R* and α. In particular, when the bottom supports are located symmetrically to the crack line (i.e., when *S*_1_ = *S*_2_) and the crack line is parallel to the load (i.e., when α = 0°), the specimen is subjected to pure opening mode I. With different combinations of *S*_2_/*R* and α, mixed-mode I/II can be obtained. In Figure 1b, a different test configuration used to determine the pure indentation behavior is shown.

Following the approach adopted in [4], the crack-like notch was initiated in two different ways: 12 samples had the notch embedded during the additive manufacturing process (named AM specimens), while the other 3 samples had the notch initiated manually by a razor blade (named SM specimens, in which SM stands for subtractive manufacturing). The AM specimens were manufactured with an angle *α* of 0° and 10° and tested, changing the support spans *S_2_*, while *S_1_* was kept at 42 mm for all the tests to obtain different mode I/II mixity. The AM specimens with crack-like notch angle *α* of 0° were only tested with symmetric configuration (pure mode I). Table 2 presents the specimens tested along with their typology, the values of α; the supports spans *S_1_* and *S_2_*, the number *n* of tested specimens and the mixed-mode ratio calculated as $M^{e}=2/\pi \mathrm{atan}\left(K_{I}/K_{II}\right)$.

### 2.3. Experimental Setup

**Figure 2:**
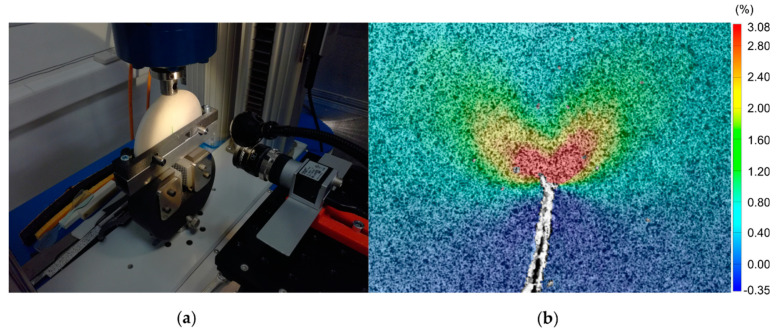
(**a**) Experimental setup; (**b**) y-strain field around the crack tip obtained by the digital image correlation (DIC) for a 10–42–42 test configuration.

Displacement controlled fracture tests were performed by using a hydraulic tensile machine. The experimental setup is shown in Figure 2a. The 2D DIC system used to evaluate the full displacement field around the crack tip (in a window of 12 mm × 9 mm) consisted of a 10 MP Basler ace acA3800-14uc camera (Basler, Ahrensburg, Germany) equipped with Basler lens C125-2522–5 M-P f25 mm (Basler, Ahrensburg, Germany) and a custom led lamp. The images were acquired by exploiting the GOM Snap 2D free software and processed by GOM Correlate (GOM GmbH, Braunschweig, Germany). A black speckle pattern was spray-painted on the specimens (which naturally exhibits a white surface), as required by the digital correlation algorithm.

The DIC parameters were set according to the study of Palanca et al. [27], as follows: 19 pixels facet size, 16 pixels point distance, 8 facets spatial filter (median) and 3 time-step temporary filters (median). This parameter combination allows obtaining an optimal compromise between the spatial resolution and the precision of the displacement field measurement around the crack tip. Figure 2b shows an example of a strain field correlated by the DIC software (y-strain, 10–42–42 test configuration).

### 2.4. J-Integral Computation via the LDC Method

As regards the IASCB specimens, the geometry factors necessary for the computation of the J-integral by the load–displacement curve method are not available in the literature, to the best of the authors’ knowledge. Therefore, the geometry factors were numerically computed for different specimen configurations in order to study the mode I-II mixity ranging from pure mode I to pure mode II. The geometry factors were then used to calculate the J-integral for the polyamide specimens by exploiting the experimental load–displacement curves (LDC method).

#### 2.4.1. FEM Calculation of the J-Integral Geometry Factors

**Figure 3:**
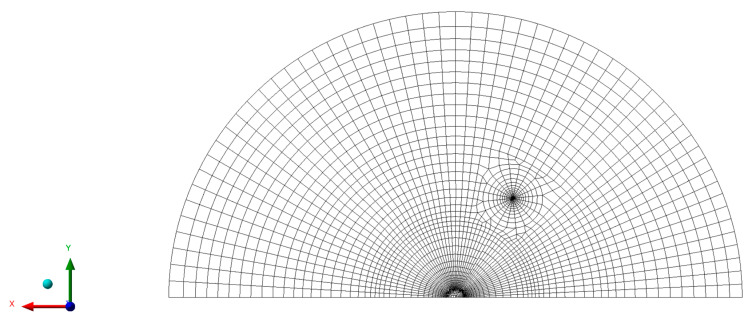
Mesh of the specimen modeled with the finite element analysis (FEM) software.

The J-integral geometry factors were computed by using the finite element commercial software Ansys 2019 (Ansys Inc., Canonsburg, Pennsylvania, USA). The IASCB specimen was discretized in approximately 4000 quadratic eight-node plane strain elements (PLANE 183), as shown in Figure 3. Since a high stress-gradient exists around the crack tip, the surrounding area was discretized with a fine radial mesh. The geometry factors are independent of the applied load, specimen thickness and material properties. Considering the arbitrariness of these factors, the simulation was run with a vertical load of 1 kN, the specimen thickness *t* was set equal to 1 mm, and the material was modeled linear elastic, with Young’s modulus *E* equal to 200 GPa and a Poisson ratio *ν* equal to 0.3. The J-integral was evaluated by the FEM software, which exploits a function based on the domain integral method proposed by Shih [28]. Six contours surrounding the crack tip were considered, and the result obtained from the last one was the one considered (results converged after three contours).

By exploiting the results obtained from the FEM analysis, the dimensionless geometry factors can be computed according to Equation (1), similarly to the standard ASTM D 6068-96 for CT and SEB specimens [20].

(1) $$\eta =\frac{J_{FEM}}{U_{FEM}}\;t\;\left(R-a\right)$$

where $J_{FEM}$ is the J-integral, expressed in mJ/mm^2^, and $U_{FEM}$ is the work done by the external load, expressed in mJ, both evaluated by the FEM software.

#### 2.4.2. J-Integral Computation from the Load–Displacement Curve Results

The geometry factors were then used to calculate the J-integral for the polyamide specimens by exploiting the experimental load–displacement curves, according to Equation (2):

(2) $$J=\eta \;\frac{U}{t\;\left(R-a\right)}$$

where *U* is the energy required to extend the crack and is obtained by subtracting to the total energy *U_T_* the indentation energy *U_i_*. The total energy is obtained by integrating the load–displacement curve up to the load at which the crack was visually observed to propagate of 1 mm, on the images acquired by the camera used for the DIC. The indentation energy *U_i_* was obtained by integrating the load–displacement curve measured in the indentation test up to the displacement that corresponds to the maximum load at which the total energy *U_T_* was evaluated. The indentation test was carried out on a specimen with the same geometry of the reference one but without the notch and an adjacent central support span, as described in Figure 1b.

### 2.5. J-Integral Computation via the DIC Method

J-integral represents the strain energy release rate or energy per unit fracture surface area of a material. It is mathematically defined as a line-integral, as shown in Equation (3):

(3) $$J=\int_{\Gamma }^{}\left(Wdy-t_{i}\frac{\partial u_{i}}{\partial x}ds\right)$$

where Γ is the integration path, W is the strain energy density, $t=\sigma \hat{n}$ is the surface traction vector ($\hat{n}$ is normal to the curve Γ and σ is the Cauchy stress tensor). One property of the J-Integral is that it does not depend on the path considered. Since the J-integral is to be evaluated starting from the DIC data, which are defined on a rectangular grid, the definition of the J-Integral is to be adapted to the case of a discrete domain. If the spacing between the grid points is low enough, this is an acceptable approximation, and it is an approach followed, for example, in [24,25,26]. The integration can be carried out by using the trapezoidal rule. For what concerns the integrand function, henceforth referred to as *f*, in the discrete domain can be rewritten as shown in Equation (4), being Δl the path increment:

(4) $$f\Delta l=W\Delta y-t_{i}\frac{\Delta u_{i}}{\Delta x}\Delta s$$

Expanding the term of the traction vector, considering the hypothesis of plane stress, and expanding the summation on the index i leads to Equation (5):

(5) $$f\Delta l=W\Delta y-\left[\frac{\Delta u_{x}}{\Delta x}\left(n_{x}\sigma_{x}+n_{y}\tau_{xy}\right)+\frac{\Delta u_{y}}{\Delta x}\left(n_{y}\sigma_{y}+n_{x}\tau_{xy}\right)\right]\Delta s$$

**Figure 4:**
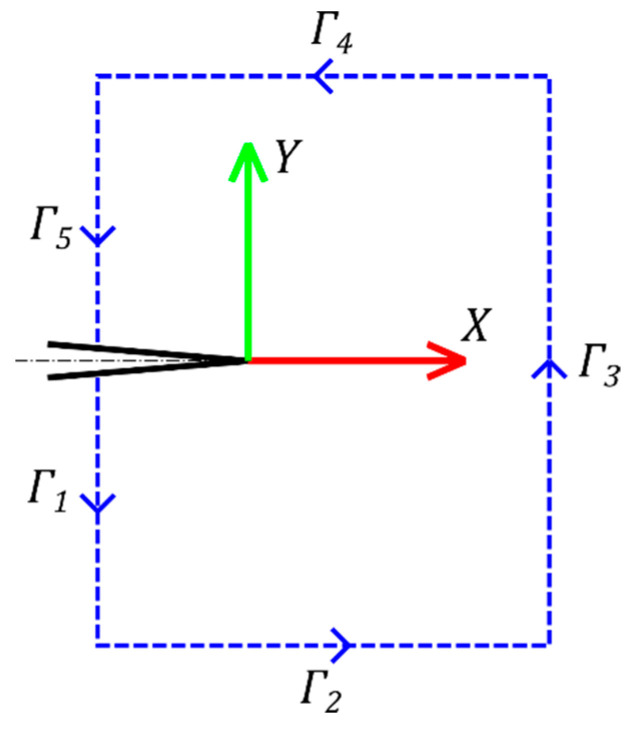
Rectangular path around the crack tip.

By breaking the rectangular path into five parts, each part can be further simplified, as shown in Equations (6)–(9) and referring to Figure 4.

Path *Γ_1_* and Path *Γ_5_*: $\hat{n}=\left(-1,0\right),\Delta s=-\left|\Delta gy\right|,\;\Delta y=-\left|\Delta gy\right|$ where |Δgy| is the distance (constant and ≥0) between 2 points of the DIC grid along the y-direction:

(6) $$f\Delta l=\left(-W-\frac{\Delta u_{x}}{\Delta x}\sigma_{x}-\frac{\Delta u_{y}}{\Delta x}\tau_{xy}\right)\left|\Delta gy\right|$$

Path *Γ_2_*: $\hat{n}=\left(0,-1\right),\Delta s=\left|\Delta gx\right|,\;\Delta y=0\;$ where |Δgx| is the distance (constant and ≥0) between two points of the DIC grid along the x-direction:

(7) $$f\Delta l=\left(\frac{\Delta u_{x}}{\Delta x}\tau_{xy}+\frac{\Delta u_{y}}{\Delta x}\sigma_{y}\right)\left|\Delta gx\right|$$

Path Γ_3_: $\hat{n}=\left(+1,0\right),\Delta s=\left|\Delta gy\right|,\Delta y=\left|\Delta gy\right|$

(8) $$f\Delta l=\left(+W-\frac{\Delta u_{x}}{\Delta x}\sigma_{x}-\frac{\Delta u_{y}}{\Delta x}\tau_{xy}\right)\left|\Delta gy\right|$$

Path Γ_4_: $\hat{n}=\left(0,+1\right),\Delta s=-\left|\Delta gx\right|,\Delta y=0$

(9) $$f\Delta l=\left(\frac{\Delta u_{x}}{\Delta x}\tau_{xy}+\frac{\Delta u_{y}}{\Delta x}\sigma_{y}\right)\left|\Delta gx\right|$$

To compute *f* in each path, the stress components are needed.

The stress components are obtained from the strain components using the elastic-plastic constitutive relations. To model the stress–strain relation, the Ramberg–Osgood equation (Equation (10)) is used:

(10) $$\frac{\varepsilon_{e}}{\varepsilon_{0}}=\frac{\sigma_{e}}{\sigma_{0}}+\alpha {\left(\frac{\sigma_{e}}{\sigma_{0}}\right)}^{n}$$

where $\sigma_{e}$ and $\varepsilon_{e}$ are the equivalent stress and strain, $\sigma_{0}$ and $\varepsilon_{0}$ (=$\sigma_{0}/E$) are the yield stress and strain, *n*, *α* and *E* the hardening exponent, the material constant and the elastic modulus, respectively. The equivalent Von Mises strain can be computed as shown in Equation (11). The strain along the *z*-direction can be computed according to Hooke’s law in the case of plain stress from the *x* and *y* components as $\varepsilon_{z}=\frac{-\nu }{\left(1-\nu \right)}\left(\varepsilon_{x}+\varepsilon_{y}\right)$. Since the tangential stresses are null on the plane, *ε_xz_* and *ε_yz_* are equally null:

(11) $$\varepsilon_{e}=\frac{2}{3}\sqrt{\varepsilon_{x}{}^{2}+\varepsilon_{y}{}^{2}+\varepsilon_{z}{}^{2}-\varepsilon_{x}\varepsilon_{y}-\varepsilon_{x}\varepsilon_{z}-\varepsilon_{y}\varepsilon_{z}+3\varepsilon_{xy}{}^{2}}$$

The Ramberg–Osgood parameters α, *n* and $\sigma_{0}$ can be obtained by an experimental characterization of the material: for that purpose, a series of three-point bending tests were performed, and Equation (10) was then fitted on the stress–strain curves experimentally measured. With these parameters available, it is then possible to compute the equivalent stress $\sigma_{e}$ by using Equation (10) and by exploiting the equivalent strain $\varepsilon_{e}$.

Following the approach used in [23], the constitutive relation can be written as shown in Equation (12):

(12) $$\varepsilon_{ij}=\frac{1+\nu }{E}s_{ij}+\frac{1-2\nu }{3E}\sigma_{kk}\delta_{ij}+\frac{3}{2}\alpha \varepsilon_{0}{\left(\frac{\sigma_{e}}{\sigma_{0}}\right)}^{n-1}\frac{s_{ij}}{\sigma_{0}}$$

where ν, $\delta_{ij}$ and $s_{ij}$ are the Poisson’s ratio, Kronecker delta and the stress deviator, respectively. The stress deviator is defined as shown in Equation (13):

(13) $$s_{ij}=\sigma_{ij}-\frac{\sigma_{kk}}{3}\delta_{ij}$$

After computing the components of the Cauchy stress tensor by exploiting Equations (10)–(13), the strain energy density *W* can be computed, according to [23], as shown in Equation (14):

(14) $$W=\;\frac{1}{E}\left[\frac{1}{2}\sigma_{e}{}^{2}+\frac{1}{2}\left(1-2\nu \right)\left(\sigma_{x}\sigma_{y}-\tau_{xy}{}^{2}\right)+\frac{\alpha n}{n+1}\sigma_{e}{}^{2}{\left(\frac{\sigma_{e}}{\sigma_{0}}\right)}^{n-1}\right]$$

## 3. Results

### 3.1. J-Integral Results from the LDC Method

**Figure 5:**
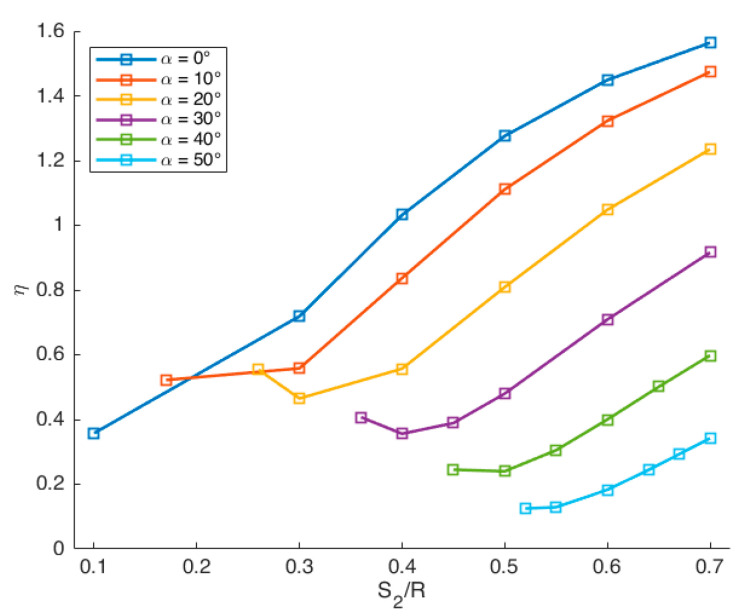
Geometry factor *η* as a function of the ratio *S*_2_*/R* for different crack angles α.

A complete set of geometry factors were calculated by the FEM model described in Section 2.4.1, for the configuration with *a/R* = 0.4 and *S_1_/R* = 0.7, by varying the support span *S*_2_ and the crack angle α. In this way, it was possible to cover the mode I-II mixity, ranging from pure mode I to pure mode II. The results are reported in Table 3, and the geometry factors are plotted in Figure 5 as a function of the *S*_2_*/R* for different crack angles. As is clear from Figure 5, η increases as the *S_2_* support span increases, while for a constant value of *S*_2_*/R*, η decreases for higher values of crack angle.

**Figure 6:**
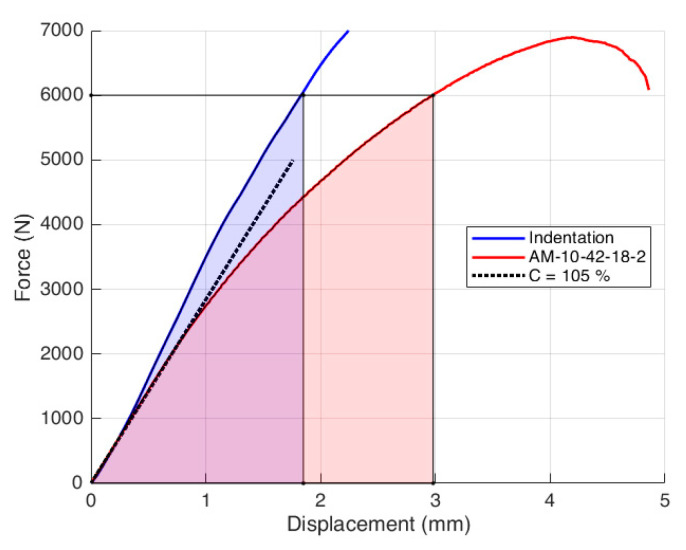
Fracture test curve (red), indentation curve (blue) and compliance line (black dotted line) in the case of the AM-10-42-18-2 test configuration.

The geometry factors were then used along with the experimental load–displacement curves to compute the J-integral. An example of the load–displacement curve for the additive manufactured specimen with a crack angle α of 10° and support span *S*_1_ = 42 mm and *S*_2_ = 18 mm (AM-10-42-18) is reported in Figure 6. The red curve represents the notched specimen, and the blue curve the unnotched one; the latter used to evaluate the indentation energy *U_i_*. The black dotted curve represents the 5% augmented tangent line with respect to the initial slope of the notched specimen, which is normally used for the stress intensity factor calculation. In the case study, however, the maximum load for the notched specimen curve is higher than 10% of its intersection with the dotted tangent line, which makes this method invalid according to the ASTM D5045-14 standard [29]. For this reason, the crack propagation start was visually evaluated by examining the frames recorded by the DIC camera. In compliance with the standard ASTM D6068-96 [20], the crack was considered to start to propagate when it reached an extension of 1 mm. The load corresponding to that time instant was then read from the load–displacement curve (roughly 6000 N in the case of Figure 6). The notched and the indentation curves are then integrated up to this load to compute the energy *U_T_* and *U_i_*, respectively, used for the calculation of the J-integral according to Equation (2).

### 3.2. J-Integral Computation via the DIC-Based Method

In this section, the results of the J-integral obtained by the DIC-based and LDC methods are presented and compared.

**Figure 7:**
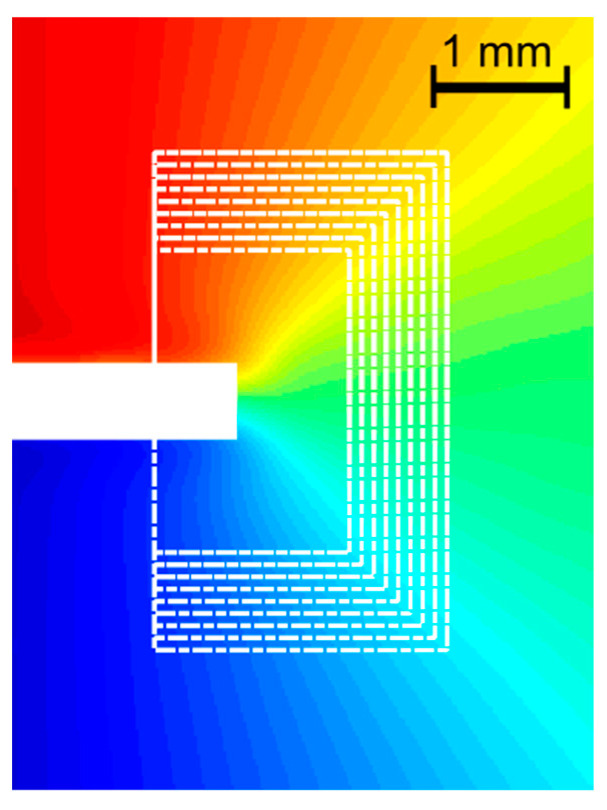
Paths considered in the computation of the J-integral.

**Figure 8:**
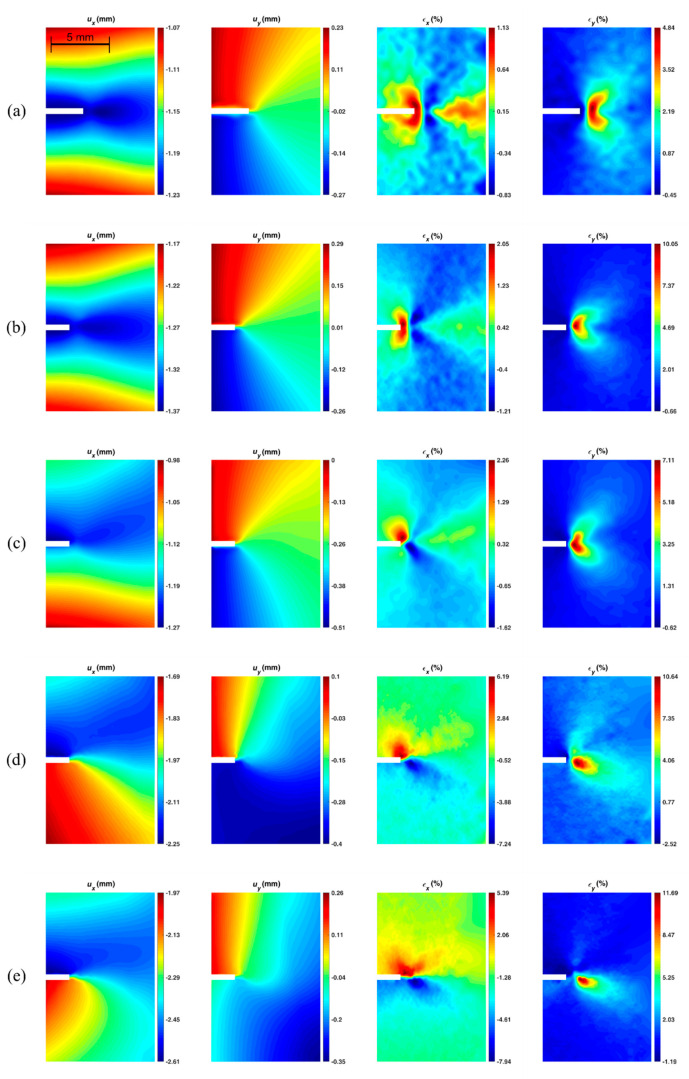
The x and y displacement (*u*) strain (*ε*) maps for different test configuration: (**a**) SM-0–42–42 (mode I); (**b**) AM-0–42–42 (mode I); (**c**) AM-10–42–42; (**d**) AM-10–42–18; (**e**) AM-10–42–10.2 (mode II).

Figure 7 shows the path around the crack tip considered for the J-integral computation. The points belonging to the zone within 0.3 mm from the crack axis were removed since, in that region, the data were particularly noisy. In this region, in fact, due to the massive plasticization and to the crack opening, the digital image correlation algorithm performs poorly. The value of 0.3 mm was empirically defined based on a visual evaluation of the measurement noise and the performance of the correlation algorithm in the proximity of the crack boundaries. An example in which a region near the crack tip is similarly removed can be found, for instance, in [30]. Figure 8 shows the x and y displacement and strain maps obtained by the DIC for all the different specimen configurations tested. It can be observed that the strain in y and in particular in x-direction increases ranging from mode I to mode II, which implies a higher strain energy work to make the crack propagate.

**Figure 9:**
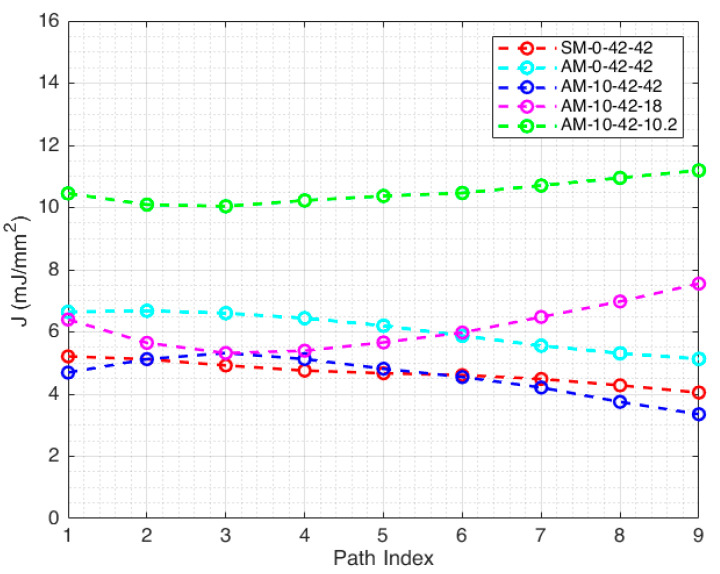
J-integral on different paths for the various specimen configurations. The lower the index, the shorter the path (referring to Figure 4).

Figure 9 shows the values of the J-integral for one specimen of each configuration on the different paths shown in Figure 7. The path index identifies the path, being the lower, the shorter path. A certain variability (up to 30%) of the J-integral can be observed on the different paths considered. The uncertainty can be attributed to two main reasons. The first one is the noise in the displacement and strain maps (particularly in these latter, due to numerical differentiation) obtained by the DIC. The second one is that the strain and the stress occurring in the proximity of the crack tip cannot be truly represented by the material model (Ramberg–Osgood) and the Poisson ratio used (which was considered constant) implemented in the J-integral model. However, the variability of the J-integral computation can be mitigated by adjusting the DIC spatial and temporal filtering and by averaging the J-integral values along different paths, similarly to [25]. As suggested in [31], it still can be used as a fracture toughness parameter, giving that it is computed over a larger domain than just a contour. For this reason, the J-integral was computed as the average of the J-Integral obtained from the contours shown in Figure 7.

## 4. Discussion

**Figure 10:**
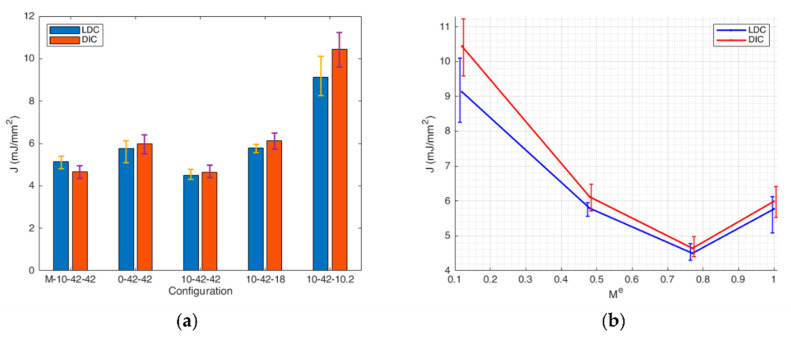
Comparison between the results obtained with the DIC method (blue) and the ones obtained with the LDC method (red). (**a**) Bar plot for the different test configurations; (**b**) J-integral plotted as a function of the mode mixity ratio *M^e^*.

In Table 4, the J-integral computed by the LDC and the DIC-based methods for different mode mixity is summarized. Figure 10 shows a comparison of the mean values obtained by the two methods. In Figure 10a the comparison is made by a bar plot, whereas Figure 10b has the mode mixity ratio *M^e^* as the x-axis.

It can be observed that the difference between the values computed by the DIC-based method and the conventional LDC method (calculated as (DIC-LDC)/LDC*100) is comparable to the experimental errors of each method (calculated as the standard deviation of the three values obtained for each configuration). This can be visually observed in Figure 10b, in which the error bands of the graphs of the two methods overlap to a significant extent.

Concerning the notch induction by the conventional subtractive method (by razor blade incision) or by additive method (by directly printing it), some considerations can be made. The J-integral mean value of the notch induced by the AM method is 12.2% higher than the one obtained by the SM method, but it is within the experimental error. This can be attributed to a less sharp crack-like notch in the case of AM specimens, which leads to a lower stress concentration in the area near the crack tip, and therefore to a higher apparent fracture toughness value.

By plotting the J-integral as a function of the mode mixity ratio *M^e^*, it can be observed that the curve has a minimum at a mixity ratio equal to 0.77 and then it increases up to pure mode II. Generally, in the literature [4], higher fracture toughness values are experimentally observed for mode II with respect to mode I. This is due to a wider stress state distribution for mode II than mode I, which leads to higher fracture toughness values.

## 5. Conclusions

In this work, the mixed-mode I/II J-integral was evaluated for polyamide ASCB specimens manufactured by the additive SLS process. A non-conventional method to produce crack-like notches during the manufacturing process, by embedding them in the geometrical modeling phase, was investigated and validated (the difference of the results in mode I of about 12%). The capability of manufacturing cracks with additive techniques is a powerful tool that can be useful for investigating the fracture mechanics behavior in the case of complex crack geometry, even three-dimensional and/or inside the structure.

Two different methods for evaluating the J-integral were compared: the conventional one based on the load–displacement curve and a less conventional one based on the evaluation of the J-integral contour by exploiting the measured full-field displacement and strain by DIC. The results obtained with this latter method were statistically comparable with the ones obtained with the former method, and the DIC method was thus demonstrated to be a valid option for investigating the fracture behavior in the case of sintered cracks on elastic–plastic material. The DIC methodology has the advantage of being contactless and can be used to evaluate the J-integral in a condition where it is not possible to use the conventional LDC method due to complex and often not known loading conditions of the structure. By combining the DIC methodology with the AM manufactured cracks, it is, therefore, possible to lay the foundations for an investigation approach that is more flexible than the standard one. Moreover, it can be easily implemented and be applied to a wider number of practical cases and real-world scenarios.

## Figures and Tables

**Table 1:** Mechanical properties of the polyamide (PA) 2200 powder.

Property	Value
Flexural Young’s modulus	2.1 ± 0.1 GPa
Flexural Yield strength	55 ± 3 MPa
Flexural strength	68 ± 2 MPa

**Table 2:** Geometrical parameters of the test configurations.

Type	α (°)	*S*_1_ (mm)	*S*_2_ (mm)	*n*	Mode	*M^e^*
SM	0	42	42	3	Mode I	1
AM	0	42	42	3	Mode I	1
AM	10	42	42	3	Mixed I/II	0.77
AM	10	42	18	3	Mixed I/II	0.48
AM	10	42	10.2	3	Mode II	0.12

**Table 3:** Geometry factor *η* for different values of *S_2_/R* and crack angle α obtained with values of *a/R* and *S_1_/R* fixed, respectively, to 0.4 and 0.7.

*S*_2_/*R*	*η*	*S_2_*/*R*	*η*	*S*_2_ */R*	*η*	*S*_2_ */R*	*η*	*S*_2_ */R*	*η*	*S*_2_ */R*	*η*
α = 0°		α = 10°		α = 20°		α = 30°		α = 40°		α = 50°	
0.7	1.564	0.7	1.474	0.7	1.235	0.7	0.916	0.7	0.598	0.7	0.342
0.6	1.449	0.6	1.323	0.6	1.048	0.6	0.709	0.65	0.501	0.67	0.293
0.5	1.276	0.5	1.111	0.5	0.809	0.5	0.480	0.6	0.400	0.64	0.244
0.4	1.032	0.4	0.837	0.4	0.557	0.45	0.389	0.55	0.305	0.6	0.183
0.3	0.719	0.3	0.558	0.3	0.465	0.4	0.356	0.5	0.240	0.55	0.129
0.1	0.358	0.17	0.522	0.26	0.554	0.36	0.407	0.45	0.245	0.52	0.125

**Table 4:** Results of the J-integral computed with the load–displacement curves (LDC) method and with the DIC method.

Specimen Configuration	M^e^	LDC Method J (mJ/mm^2^)	J_mean_	σ(J)	DIC Method J (mJ/mm^2^)	J_mean_	σ(J)	LDC vs. DIC Δ%
SM-0-42-42-1	1	5.40	5.14	0.29	4.95	4.66	0.30	−8.33
SM-0-42-42-2		4.82			4.68			−2.90
SM-0-42-42-3		5.20			4.36			−16.15
AM-0-42-42-1	1	5.09	5.77	0.59	6.42	5.99	0.45	26.13
AM-0-42-42-2		6.12			5.52			−9.80
AM-0-42-42-3		6.10			6.03			−1.15
AM-10-42-42-1	0.77	4.30	4.50	0.25	4.98	4.64	0.30	15.81
AM-10-42-42-2		4.78			4.55			−4.81
AM-10-42-42-3		4.42			4.40			−0.45
AM-10-42-18-1	0.48	5.56	5.79	0.21	6.16	6.12	0.38	10.79
AM-10-42-18-2		5.95			6.48			8.91
AM-10-42-18-3		5.87			5.72			−2.56
AM-10-42-10.2-1	0.12	9.03	9.13	0.92	9.59	10.44	0.82	6.20
AM-10-42-10.2-2		8.26			10.50			27.12
AM-10-42-10.2-3		10.10			11.23			11.19

## Data Availability

The data presented in this study are available on request from the corresponding author. The data will be publicly available at the end of the Project A_Madam.
